# *anti*-Ethyl aceto­hydroximate

**DOI:** 10.1107/S1600536813022368

**Published:** 2013-08-21

**Authors:** Barbara Hachuła, Anna Polasz, Maria Nowak, Joachim Kusz

**Affiliations:** a Institute of Chemistry, University of Silesia, 14 Bankowa Street, 40-007 Katowice, Poland; b Institute of Physics, University of Silesia, 4 Uniwersytecka Street, 40-007 Katowice, Poland

## Abstract

In the crystal structure of the title compound, C_4_H_9_NO_2_, the O—H⋯N hydrogen bonds link the mol­ecules into supra­molecular chains extending along the *b-*axis direction. The conformation of the NOH group in the nearly planar (r.m.s. deviation = 0.0546 Å) ethyl aceto­hydroximate mol­ecule is *trans* to N=C.

## Related literature

For related structures, see: Kjaer *et al.* (1977 ▶); Larsen (1971 ▶). For studies of the IR spectra of hydrogen bonding in oxime derivatives, see: Flakus *et al.* (2012 ▶). For typical bond distances, see: Allen *et al.* (1987 ▶). For hydrogen-bond motifs, see: Bernstein *et al.* (1995 ▶); Etter *et al.* (1990 ▶).
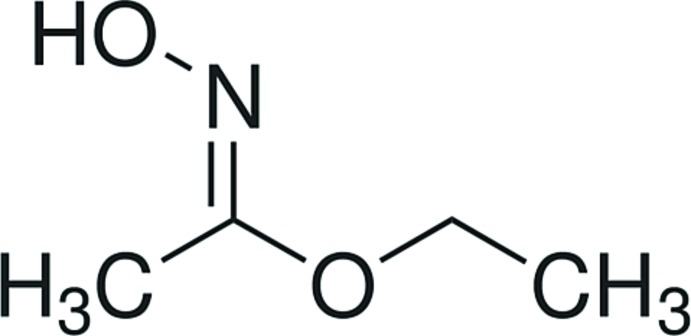


## Experimental

### 

#### Crystal data

C_4_H_9_NO_2_
*M* *_r_* = 103.12
Monoclinic, 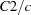

*a* = 19.9481 (9) Å
*b* = 4.4138 (1) Å
*c* = 13.3277 (5) Å
β = 109.027 (4)°
*V* = 1109.35 (7) Å^3^
*Z* = 8
Mo *K*α radiation
μ = 0.10 mm^−1^
*T* = 100 K
0.52 × 0.18 × 0.14 mm

#### Data collection

Oxford Diffraction Xcalibur diffractometer with a Sapphire3 detector
Absorption correction: multi-scan (*CrysAlis RED*; Oxford Diffraction, 2006 ▶) *T*_min_ = 0.505, *T*_max_ = 1.000
6699 measured reflections
970 independent reflections
868 reflections with *I* > 2σ(*I*)
*R*_int_ = 0.025

#### Refinement

*R*[*F* ^2^ > 2σ(*F* ^2^)] = 0.034
*wR*(*F* ^2^) = 0.098
*S* = 1.08
970 reflections
69 parameters
H atoms treated by a mixture of independent and constrained refinement
Δρ_max_ = 0.22 e Å^−3^
Δρ_min_ = −0.22 e Å^−3^

### 

Data collection: *CrysAlis CCD* (Oxford Diffraction, 2006 ▶); cell refinement: *CrysAlis CCD*; data reduction: *CrysAlis RED* (Oxford Diffraction, 2006 ▶); program(s) used to solve structure: *SHELXS97* (Sheldrick, 2008 ▶); program(s) used to refine structure: *SHELXL97* (Sheldrick, 2008 ▶); molecular graphics: *Mercury* (Macrae *et al.*, 2006 ▶); software used to prepare material for publication: *publCIF* (Westrip, 2010 ▶).

## Supplementary Material

Crystal structure: contains datablock(s) I. DOI: 10.1107/S1600536813022368/ff2116sup1.cif

Structure factors: contains datablock(s) I. DOI: 10.1107/S1600536813022368/ff2116Isup2.hkl

Supplementary material file. DOI: 10.1107/S1600536813022368/ff2116Isup3.cml

Additional supplementary materials:  crystallographic information; 3D view; checkCIF report

## Figures and Tables

**Table 1:** Hydrogen-bond geometry (Å, °)

*D*—H⋯*A*	*D*—H	H⋯*A*	*D*⋯*A*	*D*—H⋯*A*
O1—H1⋯N1^i^	0.871 (19)	1.954 (19)	2.8196 (14)	172.4 (16)

Symmetry code: (i) 
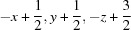
.

**Fig. 1.**
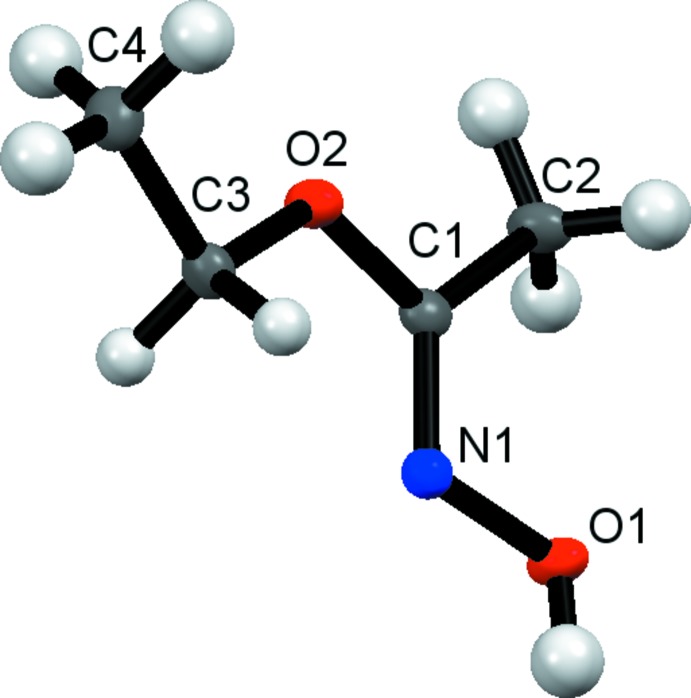
The asymmetric unit of (I), with the atom-numbering scheme, showing 50% probability displacement ellipsoids. H atoms are shown as small spheres of arbitrary radius.

**Fig. 2.**
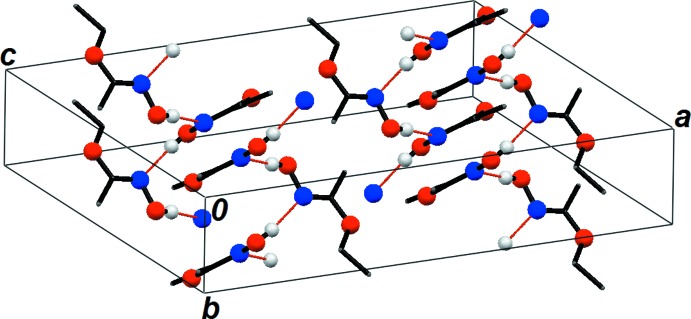
Part of the crystal structure of (I), showing the C(3) chains. The red lines indicate the hydrogen-bonding interactions. For the sake of clarity, all H atoms bonded to C atoms were omitted.

## supplementary crystallographic information

### 1. Comment

*Anti*-ethyl acetohydroximate [systematic name: ethyl *N*-hydroxyacetimidate], (I), was investigated in a continuation of our studies of the IR spectra of hydrogen bonding in oxime derivatives (Flakus *et al.*, 2012). In order to study interactions occurring *via* hydrogen bonds and molecular packing in this compound, we have now determined the structure of (I) using diffraction data collected at 100 K. Until now, the structures of *syn*-methyl acetohydroximate and *syn*- and *anti*-ethyl benzohydroximate were determined (Kjaer *et al.*, 1977; Larsen *et al.*, 1971). The crystal structure of *syn*-methyl acetohydroximate is composed of layers of molecules, which form cyclic, hydrogen-bonded trimers, whereas the crystals of *syn*- and *anti*-ethyl benzohydroximate are composed of dimers formed by pairs of O—H···N hydrogen- bonded molecules.

The molecule of (I) is nearly planar (r.m.s. deviations 0.0546 Å for all non-H atoms). The lengths of the bonds C=N (1.2771 (17) Å) and N—O (1.4286 (13) Å) in (I) are comparable to the mean values found in other oximes (C=N 1.281 Å; N—O 1.394 Å) (Allen *et al.*, 1987). The conformation of the NOH group in the planar ethyl acetohydroximate molecule is *trans* to N=C. In the crystal, O—H···N are observed forming infinite chains along the *b* axis (Fig. 2) with a graph-set motif of *C*(3) (Etter *et al.*, 1990; Bernstein *et al.*, 1995).

### 2. Experimental

Ethyl acetohydroximate was purchased from Aldrich-Sigma. Crystals of title compound, suitable for X-ray diffraction, were selected directly from purchased sample.

### 3. Refinement

The H atoms were introduced in geometrically idealized positions with C—H distances of 0.99 Å and *U*_iso_(H) values set at 1.2*U*_eq_(C) for methylene group or 0.98 Å and with *U*_iso_(H) values set at 1.5*U*_eq_(C) for methyl groups. The H atom which takes part in hydrogen bonding was located in a difference Fourier map and was refined with *U*_iso_(H) value set at 1.5*U*_eq_(O).

### Crystal data

**Table:**

C_4_H_9_NO_2_	*F*(000) = 448
*M**_r_* = 103.12	*D*_x_ = 1.235 Mg m^−^^3^
Monoclinic, *C*2/*c*	Melting point = 296–298 K
Hall symbol: -C 2yc	Mo *K*α radiation, λ = 0.71073 Å
*a* = 19.9481 (9) Å	Cell parameters from 6376 reflections
*b* = 4.4138 (1) Å	θ = 3.1–34.4°
*c* = 13.3277 (5) Å	µ = 0.10 mm^−^^1^
β = 109.027 (4)°	*T* = 100 K
*V* = 1109.35 (7) Å^3^	Needle, colourless
*Z* = 8	0.52 × 0.18 × 0.14 mm

### Data collection

**Table:**

Oxford Diffraction Xcalibur diffractometer with a Sapphire3 detector	970 independent reflections
Radiation source: fine-focus sealed tube	868 reflections with *I* > 2σ(*I*)
Graphite monochromator	*R*_int_ = 0.025
Detector resolution: 16.0328 pixels mm^-1^	θ_max_ = 25.1°, θ_min_ = 3.2°
ω–scan	*h* = −22→23
Absorption correction: multi-scan (*CrysAlis RED*; Oxford Diffraction, 2006)	*k* = −2→5
*T*_min_ = 0.505, *T*_max_ = 1.000	*l* = −15→15
6699 measured reflections	

### Refinement

**Table:**

Refinement on *F*^2^	Primary atom site location: structure-invariant direct methods
Least-squares matrix: full	Secondary atom site location: difference Fourier map
*R*[*F*^2^ > 2σ(*F*^2^)] = 0.034	Hydrogen site location: inferred from neighbouring sites
*wR*(*F*^2^) = 0.098	H atoms treated by a mixture of independent and constrained refinement
*S* = 1.08	*w* = 1/[σ^2^(*F*_o_^2^) + (0.0616*P*)^2^ + 0.5949*P*] where *P* = (*F*_o_^2^ + 2*F*_c_^2^)/3
970 reflections	(Δ/σ)_max_ < 0.001
69 parameters	Δρ_max_ = 0.22 e Å^−^^3^
0 restraints	Δρ_min_ = −0.22 e Å^−^^3^

### Special details

**Table:**

Geometry. All e.s.d.'s (except the e.s.d. in the dihedral angle between two l.s. planes) are estimated using the full covariance matrix. The cell e.s.d.'s are taken into account individually in the estimation of e.s.d.'s in distances, angles and torsion angles; correlations between e.s.d.'s in cell parameters are only used when they are defined by crystal symmetry. An approximate (isotropic) treatment of cell e.s.d.'s is used for estimating e.s.d.'s involving l.s. planes.
Refinement. Refinement of *F*^2^ against ALL reflections. The weighted *R*-factor *wR* and goodness of fit *S* are based on *F*^2^, conventional *R*-factors *R* are based on *F*, with *F* set to zero for negative *F*^2^. The threshold expression of *F*^2^ > σ(*F*^2^) is used only for calculating *R*-factors(gt) *etc*. and is not relevant to the choice of reflections for refinement. *R*-factors based on *F*^2^ are statistically about twice as large as those based on *F*, and *R*- factors based on ALL data will be even larger.

### Fractional atomic coordinates and isotropic or equivalent isotropic displacement parameters (Å^2^)

**Table:**

	*x*	*y*	*z*	*U*_iso_*/*U*_eq_	
O1	0.31644 (5)	0.6668 (2)	0.82875 (7)	0.0201 (3)	
O2	0.39491 (4)	0.1706 (2)	0.69431 (7)	0.0185 (3)	
N1	0.31725 (5)	0.4501 (2)	0.74954 (8)	0.0165 (3)	
C1	0.38102 (7)	0.3704 (3)	0.76216 (10)	0.0158 (3)	
C2	0.44623 (7)	0.4799 (3)	0.84598 (10)	0.0215 (3)	
H2A	0.4568	0.6871	0.8294	0.032*	
H2B	0.4862	0.3472	0.8486	0.032*	
H2C	0.4385	0.4774	0.9149	0.032*	
C3	0.33586 (7)	0.0728 (3)	0.60358 (10)	0.0187 (3)	
H3A	0.3103	0.2503	0.5636	0.022*	
H3B	0.3022	−0.0497	0.6271	0.022*	
C4	0.36699 (8)	−0.1145 (3)	0.53500 (11)	0.0235 (3)	
H4A	0.3987	0.0118	0.5100	0.035*	
H4B	0.3287	−0.1929	0.4739	0.035*	
H4C	0.3938	−0.2843	0.5765	0.035*	
H1	0.2735 (10)	0.740 (4)	0.8065 (13)	0.035*	

### Atomic displacement parameters (Å^2^)

**Table:**

	*U*^11^	*U*^22^	*U*^33^	*U*^12^	*U*^13^	*U*^23^
O1	0.0168 (5)	0.0243 (5)	0.0190 (5)	0.0038 (4)	0.0054 (4)	−0.0041 (4)
O2	0.0153 (5)	0.0230 (5)	0.0163 (5)	0.0012 (3)	0.0041 (4)	−0.0017 (4)
N1	0.0170 (6)	0.0172 (6)	0.0154 (6)	−0.0001 (4)	0.0052 (4)	0.0006 (4)
C1	0.0167 (7)	0.0174 (6)	0.0140 (6)	0.0000 (5)	0.0062 (5)	0.0034 (5)
C2	0.0152 (7)	0.0290 (7)	0.0193 (7)	0.0011 (5)	0.0042 (5)	−0.0014 (6)
C3	0.0175 (7)	0.0201 (7)	0.0164 (7)	−0.0019 (5)	0.0026 (5)	0.0002 (5)
C4	0.0282 (7)	0.0241 (7)	0.0188 (7)	−0.0025 (6)	0.0086 (6)	−0.0006 (5)

### Geometric parameters (Å, º)

**Table:**

O1—N1	1.4286 (13)	C2—H2C	0.9800
O1—H1	0.871 (19)	C3—C4	1.5082 (17)
O2—C1	1.3547 (15)	C3—H3A	0.9900
O2—C3	1.4517 (15)	C3—H3B	0.9900
N1—C1	1.2771 (17)	C4—H4A	0.9800
C1—C2	1.4922 (17)	C4—H4B	0.9800
C2—H2A	0.9800	C4—H4C	0.9800
C2—H2B	0.9800		
			
N1—O1—H1	104.1 (11)	O2—C3—C4	106.57 (10)
C1—O2—C3	117.55 (9)	O2—C3—H3A	110.4
C1—N1—O1	109.76 (10)	C4—C3—H3A	110.4
N1—C1—O2	120.21 (12)	O2—C3—H3B	110.4
N1—C1—C2	126.66 (12)	C4—C3—H3B	110.4
O2—C1—C2	113.11 (10)	H3A—C3—H3B	108.6
C1—C2—H2A	109.5	C3—C4—H4A	109.5
C1—C2—H2B	109.5	C3—C4—H4B	109.5
H2A—C2—H2B	109.5	H4A—C4—H4B	109.5
C1—C2—H2C	109.5	C3—C4—H4C	109.5
H2A—C2—H2C	109.5	H4A—C4—H4C	109.5
H2B—C2—H2C	109.5	H4B—C4—H4C	109.5
			
O1—N1—C1—O2	−178.49 (9)	C3—O2—C1—C2	−174.04 (10)
O1—N1—C1—C2	0.24 (17)	C1—O2—C3—C4	173.10 (10)
C3—O2—C1—N1	4.85 (16)		

### Hydrogen-bond geometry (Å, º)

**Table:**

*D*—H···*A*	*D*—H	H···*A*	*D*···*A*	*D*—H···*A*
O1—H1···N1^i^	0.871 (19)	1.954 (19)	2.8196 (14)	172.4 (16)

Symmetry code: (i) −*x*+1/2, *y*+1/2, −*z*+3/2.
